# Natural Diversity of Cuticular Pheromones in a Local Population of Drosophila after Laboratory Acclimation

**DOI:** 10.3390/insects15040273

**Published:** 2024-04-15

**Authors:** Jean-François Ferveur, Jérôme Cortot, Matthew Cobb, Claude Everaerts

**Affiliations:** 1 Centre des Sciences du Goût et de l’Alimentation, Unité Mixte de Recherche 6265 Centre National de la Recherche Scientifique, Unité Mixte de Recherche 1324 Institut National de la Recherche Agronomique, Université de Bourgogne Franche-Comté, 6, Bd Gabriel, 21000 Dijon, France; jerome.cortot@u-bourgogne.fr (J.C.); claude.everaerts@u-bourgogne.fr (C.E.); 2 School of Biological Sciences, University of Manchester, Manchester M13 9PT, UK; cobb@manchester.ac.uk

**Keywords:** 5,9-pentacosadiene, 7-pentacosene, 5,9-heptacosadiene, 7-tricosene

## Abstract

**Simple Summary:**

Insects are covered with chemicals called hydrocarbons that are involved in chemical communication (mainly in mating) as well as in preventing the insect from drying out and protecting it from harmful environmental factors. Although we know a great deal about these molecules in laboratory strains, little is known about their variation and evolution in nature. We studied *Drosophila melanogaster* flies from Dijon, France, which we captured using traps baited with various fruits. We set up lines from these flies and studied their hydrocarbons on capture and after up to 40 generations in the laboratory. We initially observed a lot of natural variability, but this soon became more limited as the strains adapted to life in the laboratory. In some of the lines, females flies had a hydrocarbon profile previously seen only in tropical populations: this may indicate that this globally distributed species is adapting to increased temperatures caused by climate change. These hydrocarbons can be used as sensitive markers of climate change, as long as they are studied before insects become adapted to laboratory conditions.

**Abstract:**

Experimental studies of insects are often based on strains raised for many generations in constant laboratory conditions. However, laboratory acclimation could reduce species diversity reflecting adaptation to varied natural niches. Hydrocarbons covering the insect cuticle (cuticular hydrocarbons; CHCs) are reliable adaptation markers. They are involved in dehydration reduction and protection against harmful factors. CHCs can also be involved in chemical communication principally related to reproduction. However, the diversity of CHC profiles in nature and their evolution in the laboratory have rarely been investigated. Here, we sampled CHC natural diversity in *Drosophila melanogaster* flies from a particular location in a temperate region. We also measured *cis*-Vaccenyl acetate, a male-specific volatile pheromone. After trapping flies using varied fruit baits, we set up 21 *D. melanogaster* lines and analysed their pheromones at capture and after 1 to 40 generations in the laboratory. Under laboratory conditions, the broad initial pheromonal diversity found in male and female flies rapidly changed and became more limited. In some females, we detected CHCs only reported in tropical populations: the presence of flies with a novel CHC profile may reflect the rapid adaptation of this cosmopolitan species to global warming in a temperate area.

## 1. Introduction

The insect cuticle is covered with a blend of long-chain hydrocarbons (cuticular hydrocarbons; CHCs); these compounds, which are necessary for survival and reproduction, constitute a multifaceted phenotype reflecting both climate adaptation and chemical communication [1,2,3,4,5,6]. A key function of CHCs in insects is the reduction in water loss due to external temperature and humidity [7,8,9,10]. These kinds of factors can be affected by global warming through adaptation to changed climatic conditions, which can affect diversity and may involve invasive events by species or strains [11,12,13,14,15,16]. These properties depend on CHC chemical structure, in particular chain length and number of double bonds [17,18]. CHCs are also involved in protection against harmful external factors (predators, microorganisms, pollutants, xenobiotics and so on) [19,20,21,22]. In many insect species, CHCs are also necessary for inter-individual communication underlying courtship and mating behaviours, territory, nest and trail marking, and aggressive interactions [23,24,25,26,27,28].

**Figure 1:**
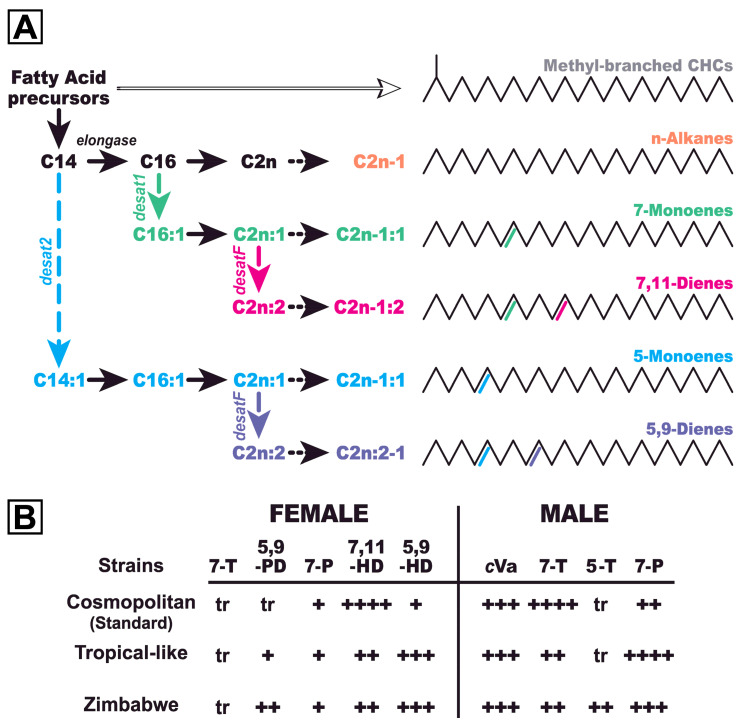
Biosynthesis and relative production of pheromones in *D. melanogaster*. (**A**) Schematic representation of the biosynthesis of cuticular hydrocarbons (CHCs) in *D. melanogaster* flies. Short-chain fatty acid precursors are combined to produce carbon chains with 14 carbon atoms (C14). C14 chains can be either desaturated by the desaturase2 enzyme (desat2) leading to C5-unsaturated CHCs or be elongated (by the addition of 2 carbon atoms with an elongase) to produce a C16 carbon chain which can be, or not, desaturated by the desaturase1 enzyme (desat1) leading to C7-unsaturated CHCs. Non-desaturated carbon chains will produce, after one or several elongation(s) steps, n-Alkanes, whereas C5- and C7-unsaturated CHCs will lead (after elongation) to 5- or 7-monoenes, respectively. In the two monoenes series, a second female-specific desaturase (desatF) can produce 5,9- and 7,11-dienes. At last, all elongated (saturated and unsaturated) carbon chains are subjected to a single decarboxylation step transforming even-number carbon chains into odd-number carbon chains which are the final CHC products. The biosynthesis of methyl-branched CHCs follows a distinct pathway. (**B**) Relative amounts of whole-body CHCs and *cis*-Vaccenyl acetate (*c*Va) in females and males of three types of strains (Cosmopolitan, Tropical-like and Zimbabwe). CHCs shown are (from left to right): 7-tricosene (7-T; 23 carbons; C7 desaturation), 5,9-pentacosadiene (5,9-PD, 25 carbons; C5 and C9 desaturations), 7-pentacosene (7-P; 25 carbons), 7,11-heptacosadiene (7,11-HD; 27 carbons), 5,9-heptacosadiene (5,9-HD) and 5- tricosene (5-T). The indications ”tr” ( = traces ≤ 5%); “+” ( = 5–10%); “++” ( = 10–20%);”+++” ( = 20–30%); “++++” (>30%) correspond to the amount of each compound relative to the total absolute amount of CHCs.

CHCs have been intensively studied in the model species *Drosophila melanogaster*. Their biosynthesis depends on an elaborate enzymatic network [25,29,30,31] that involves several molecular families including fatty-acid synthetases, elongases, desaturases and decarboxylases [32,33] (Figure 1A). The quantity of the final CHC products depends on the fine equilibrium between these enzymes, which is a function of both gene expression and precursor availability. Within each enzymatic family, these molecules show a very fine substrate specificity. For example, two closely related *D. melanogaster* desaturase enzymes, desaturase 1 and 2, act on two distinct carbon chains which differ in length by two carbon atoms, leading to C7 or C5 desaturation, respectively [33,34].

CHCs of the cosmopolitan species *D. melanogaster* show a qualitative sexual dimorphism and a marked geographic variability (Figure 1B). In temperate areas, males produce high amounts of 7-tricosene (7-T; 23 carbons; C7 desaturation) and low 7-pentacosene (7-P; 25 carbons; C7 desaturation), whereas females predominantly produce 7,11-heptacosadiene (7,11-HD; 27 carbons; C7 and C11 desaturation) [35]. In West African and Caribbean strains, males produce high levels of 7-P and low levels of 7-T [30], whereas females produce high levels of 5,9-heptacosadiene (5,9-HD; C5 and C9 unsaturations) and lower levels of 7,11-HD [36,37,38]. In strains collected in a natural park of Zimbabwe, females produce high 5,9-HD levels while males produce relatively high levels of 5-tricosene (5-T; C5 unsaturation) [39,40]. These data strongly suggest that worldwide CHC variation is related to specific climatic and environmental conditions [3,4,9]. Several studies have compared CHCs in *D. melanogaster* strains sampled on a worldwide, continental or regional scale [36,38,41,42,43]. For example, global quantitative variation of 7,11-HD in females [36] and the 7-T/7-P ratio in males have been studied [4,41], and a comparison has been made between strains from vineyards in Burgundy (France) separated by less than 45 kms shortly after collection (2002) and after many generations in the laboratory (2007); this revealed that overall CHC variability changed between strains but remained broad between the two dates [42]. In all these studies, CHC analysis was not performed on capture, but after at least a few generations in the laboratory. The sexual activity of four isofemale lines caught on banana baits separated by 50 m was compared with three laboratory long-established lines; however, female CHCs were not studied and the time since collection for the laboratory lines was not indicated [43]. An earlier study of *Drosophila mojavensis* flies sampled on banana baits apparently showed rapid CHC change under laboratory conditions [6]. However, this finding might equally be caused by a possible bottleneck effect, given that it was obtained from a single isofemale line.

**Figure 2:**
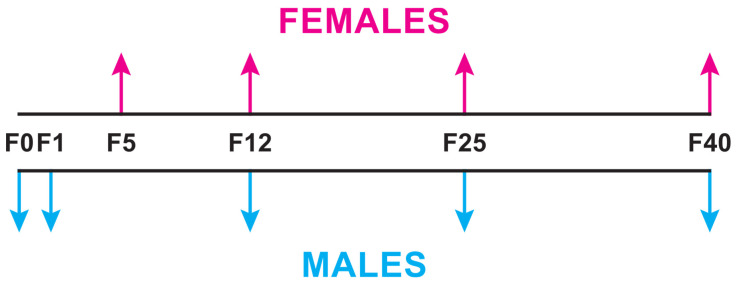
Temporal sampling procedure for chemical analysis. Arrows represent the generations (F) at which flies were sampled for chemical analysis: for females (above; at F5, F12, F25 and F40) and males (below; F0, F1, F12, F25, F40).

We evaluated CHC variability in *D. melanogaster* flies from a restricted natural area (400 square meters) by collecting flies from traps containing different types of fruit. We used different fruit baits to increase our chances of catching flies and to compare the attractiveness of the different fruits to flies that might belong to different species. All the flies caught in each trap were used to set up a line. CHCs were analysed in founder males (F0) and in F1 males; females were analysed after five generations in the laboratory (F5; Figure 2). CHCs were subsequently analysed in both sexes at F12, F25 and F40, either in individual lines or in lines grouped based on the type of fruit bait. The initial CHC variation found in both sexes evolved during laboratory acclimation. Slight but significant difference was found in relation to the fruit bait. Surprisingly, a variant—tropical-like—female CHC profile was detected in some F5 flies and was maintained until F40.

## 2. Materials and Methods

### 2.1. Flies

Sampling. In early September 2021, we set out 22 traps containing five types of fruits (fig, peach, mirabelle, banana, melon) in an orchard in Dijon, France. Traps were opened at 7 p.m. and closed at 10 p.m. on two successive days. This was the period of the day when the sun set and the temperature become cooler, allowing more flies to look for food. Together with dawn, it is one of the two periods of highest activity during the circadian cycle in *Drosophila* (‘Dew-lover’). From these traps, we set up 21 distinct lines with founder flies caught in each trap, each day (F0; Table 1; Trap #22 contained *Drosophila busckii* flies). At F1, species identity was checked by visual inspection under a binocular microscope; flies which morphologically obviously did not belong to *D. melanogaster* or to its sibling species *D. simulans* were discarded; some F1 males were set aside for chemical analysis. Crosses between Cs females and F2 males of each line were carried out to be certain that the F2 flies were *D. melanogaster* (and not *D. simulans*); this was confirmed by the presence of both male and female F3 progeny (Table 1). The sex ratio and the number of F3 male progeny were determined for each line, together with any lethality in later generations. These fitness parameters were measured to ensure that any potential CHC variation would not be caused by general physiological defects. No significant sex-ratio distortion was noted at F3, although the number of F3 males varied substantially between lines (e.g., 31 males in line #13 and 110 males in line #15). None of these lines, kept inbred and checked at each generation (on transfer to a fresh food vial), showed any major reproductive problem. Line #10 (“Peach2”) consistently showed lower fecundity over fifteen generations. To avoid bottleneck effects, each line was maintained in a minimum of six fresh food vials; this number was increased to 12–18 vials at F11 and F24 in order to have sufficient flies for CHC extractions. At F12 and F25, we also measured lethality in 4-day-old same-sex flies kept in small groups (Table 1). Female lethality varied between 0 and 32%, while male lethality varied between 0 and 8%.

Maintenance. Stocks were maintained on alcohol-free standard cornmeal medium mixed with killed yeast in 30 mL glass vials, at 24 ± 0.5 °C and 65 ± 5% humidity on a 12:12 dark/light cycle. To avoid larval competition, only 8–10 pairs of flies were kept in each vial and transferred to fresh food vials every 4–5 days or every 2–3 days when we needed to expand the stocks at F11 and F24. To isolate flies for chemical extraction, we sexed 1- to 2-hour-old flies under light carbon dioxide anaesthesia 2–4 h after lights were turned on. These flies were kept in glass vials containing fresh food in small groups of five flies and tested when 4 days old. F0 males were analysed 3 days after field collection to allow them to fertilize F0 females: they had at least 4 days.

Preparation for extraction. Males were sampled at F1, F12, F25 and F40 (Figure 2). To avoid stock depletion, virgin females from each line were first sampled at F5, then at F12, F25 and F40 generations. Both F12 and F25 analyses were performed on all lines; F40 analysis was performed on four lines, three of which had produced females with a variant CHC pattern at F25.

### 2.2. Chemical Analysis

Our analysis involved whole-body extraction: frozen flies were individually immersed for 24 h in 30 µL dichloromethane at room temperature. This procedure, successfully used in a previous study, allowed us to extract the complete amounts of CHCs and of 11-*cis*-vaccenyl acetate (*c*Va) [44]. The solvent contained 3.33 ng/µL of C26 (*n*-hexacosane) and 3.33 ng/µL of C30 (*n*-triacontane) used as internal standards (ISs). Amounts of *c*Va and CHCs were quantified by gas chromatography using a Varian CP3380 gas chromatograph fitted with a flame ionization detector, a CP Sil 5CB column (25 m × 0.25 mm internal diameter; 0.1 µm film thickness; Agilent), and a split–splitless injector (60 mL/min split-flow; valve opening 30 sec after injection) with helium as the carrier gas (50 cm/s at 120 °C). The temperature program began at 120 °C, ramping at 10 °C/min to 140 °C, then ramping at 2 °C/min to 290 °C, and holding for 10 min. The chemical identity of each peak was determined according to Everaerts et al. [45]. The amount (ng/insect) of each compound was calculated based on the readings obtained from the ISs. In some F40 females, 7-T, 7P, 7,11-HD, 5,9-PD and 5,9-HD were identified by GC-MS (see below). Each set of measures was performed several times, separated in time by a few days.

For GC-MS, a QP2010 Shimadzu GC-MS apparatus in splitless mode, fitted with a VF-1 ms fused-silica capillary column (20 m × 0.15 mm ID, 0.15 µm film thickness, Varian) was used. The column was held isothermally at 140 °C, then programmed at the rate of 3 °C/min to 300 °C. Helium was used as carrier gas at a linear velocity of 47 cm/s. The injector port was set at 280 °C. The mass spectrometer was operated at 70 eV, and scanning was performed from 29 to 600 amu at 0.5 scans/s. The injection split was opened 1 min after the injection. The detected components were identified using their Kovats indices [46]; their fragmentation patterns and diagnostic ions were compared with both the NIST/EPA/NIH library and our own mass spectrum library and compared with previously published Drosophila CHCs.

In males, we analysed 7-tricosene (7-T) and 7-pentacosene (7-P). We also measured the 7-T/7-P ratio as a way of determining the global *vs* compound-specific effect induced by laboratory acclimation. We also measured the level of *c*Va, a non-CHC compound produced by a separate biosynthetic pathway [47]. As well as revealing natural diversity in *c*Va levels and the effect of laboratory acclimation, this compound can also be considered as an external biochemical marker. In females, we analysed 7-T, 5,9-pentacosadiene (5,9-PD), 7-P, 7,11-heptacosadiene (7,11-HD) and 5,9-heptacosadiene (5,9-HD). We also determined the ratio between two pairs of closely related CHCs: 7-P/5,9-PD and 7,11-HD/5,9-HD, for similar reasons as for 7-T/7-P. Additionally, 5,9-PD, which has not been previously studied, was identified following GC-MS on F40 females. We subsequently calculated the amount of this compound on gas chromatograms of F5, F12 and F25 females.

### 2.3. Statistics

We compared the amounts and ratios of the various chemicals or ratios using the Kruskal–Wallis test with Monte Carlo simulations, followed by Conover–Iman multiple pairwise comparisons (*p* = 0.05, with Bonferroni’s correction). In females, linear regression was also used to model the possible relationship between 7-P/5,9-PD or 7,11-HD/5,9-HD ratios and generations (respectively, dependent and explanatory variables; least-squares method; slope significances were computed using Analysis of Variance—type III SS). Statistical analyses were performed using XLSTAT Premium 2021.5.1.1220 (Addinsoft 2021).

## 3. Results

### 3.1. Comparison between Males in Individual Lines

We measured and compared the mean absolute amounts (in ng) of *c*Va, 7-T, 7-P and of the 7-T/7P ratio in males of the 21 lines at F0, F1, F12, F25 and F40 generations (Supplementary Figure S1). We also highlight line(s) showing a remarkable pattern.

*c*Va. No variation was observed at F0. At F1, *c*Va varied between 1386 and 2430 ng. At F12, its range was between 827 and 1147 ng. At F25, its range was 814–1408 ng. At F40, its range was 1080–1685 ng. In summary, despite significant differences at each generation, no line showed a consistent *c*Va change across generations.

7-T. At F0, a very important inter-line difference in 7-T amount was observed, varying from 831 to 1989 ng. At F1, it varied between 1456 and 2366 ng. At F12, the range of 7-T was 584–964 ng. At F25, it ranged between 851 and 1688 ng. At F40, it varied between 1049 and 2145 ng. From F12 to F40, line #7 showed the lowest 7-T level.

7-P. At all generations, 7-P showed important variation. At F0, it varied between 78 and 600 ng; at F1, between 145 and 598 ng; at F12, the range was 164–521 ng; at F25, 7-P level varied between 189–633 ng and ng. At F40, the range was 787–175 ng. Overall, line #10 showed a constant high 7-P level between F12 and F40, while line #11 showed the lowest 7-P level at F1, F25 and F40.

7-T/7-P ratio. At F0, the ratio varied between 3.7 and 16.2. At F1, its range was 4.3–17.4. At F12, the ratio varied between 2.0 and 8.4. At F25, it varied between 2.6 and 10.0. At F40, the range was 1.9–11.9. In summary, line #11 consistently showed a high 7-T/7-P ratio from F1 to F40, while line #10 showed the lowest ratio from F12 to F40.

The variation across generations (from F0 to F40) was also studied for each line and parameter (Supplementary Figure S2). However, for the sake of simplicity, these data are not discussed here since they do not carry extra information compared to the “Fruit lines” group analysis (see below).

### 3.2. Comparison between Males in the Four “Fruit Lines” Groups

**Figure 3:**
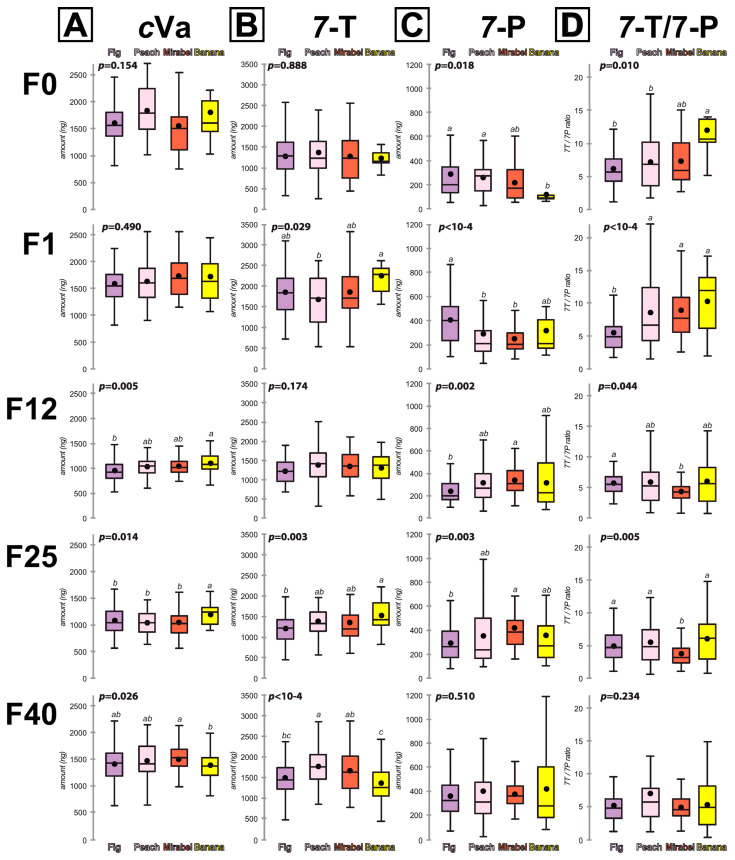
Male pheromone production in “Fruit lines” over five generations. Various pheromonal parameters were measured: (**A**) *c*Va, (**B**) 7-T, (**C**) 7-P and (**D**) the 7-T/7-P ratio. Their levels were measured at F0, F1, F12, F25 and F40. Lines were grouped according to their initial fruit preference (“Fruit lines” groups are: “Fig”, “Peach”, “Mirabel”, “Banana”; indicated beneath top and bottom bars). Data are shown as box and whisker diagrams. The lower and upper box edges represent the first and third quartiles, while the median value is indicated by the inner small horizontal bar and the mean by the plain dot. The ends of the whiskers above and below each box represent the limits beyond which values were considered anomalous. The levels of the various chemicals or ratios were compared using the Kruskal–Wallis test with Monte Carlo simulations, followed by Conover–Iman multiple pairwise comparisons (*p* = 0.05, with Bonferroni’s correction). Different letters indicate significant differences between the different groups at each generation. The statistical value of each comparison is indicated at the top left of each set. For more details on parameters, see Figure 1 legend. For more detail on sample size, see Supplementary Figure S1 legend.

We compared each pheromone parameter between lines grouped according to the kind of fruit used in the trap. We studied four groups of “Fruit lines” (“Fig”, “Peach”, “Mirabel”, “Banana”). “Melon” was not considered as a group since it was only represented by a single line (#21). For each parameter, we compared groups at each generation (Figure 3), and each group between generations (Supplementary Figure S3).

*c*Va. Between groups: No difference was observed between groups at F0 and F1 (Figure 3A). Compared to the three other groups, the “Banana” group showed the highest *c*Va level at F12 and F25 and the lowest level at F40.

Between generations: The initial *c*Va level observed in F0 and F1 in all four groups strongly decreased in F12 before increasing progressively at F15. At F40, it reached a level similar or very close to the initial F0 and F1 values (Supplementary Figure S3A).

7-T. Between groups: No important differences were observed between groups. We note that the F1 differences observed between the “Banana” group (highest value) and the “Peach” group (lowest value) were reversed at F40 (Figure 3B).

Between generations: In all four groups, the amount of 7-T increased significantly in F1 flies compared to F0 males before strongly decreasing at F12 (as with *c*Va). Subsequently, 7-T levels increased progressively at F25 and reached a level similar or very close to the F0/F1 values at F40 (Supplementary Figure S3B). 

7-P. Between groups: At F0, the “Banana” group showed the lowest level (Figure 3C). The “Fig” group showed a contrasted pattern with the highest level at F1 and the lowest one at F12 and F25. At F12 and F25, the “Mirabel” group showed the highest 7-P level. No difference was found at F40.

Between generations: In all groups, 7-P tended to increase during laboratory acclimation (from F0 to F40). Only the “Fig” group showed a slightly decreased F12 value before increasing until F40 (Supplementary Figure S3C). 

7-T/7-P ratio. Between groups: The differences observed at F0 (highest ratio in “Banana” group), at F1 (lowest in “Fig” group), and at F12 and F25 (lowest in “Mirabel” group) were not seen at F40 (Figure 3D). 

Between generations: The 7-T/7-P ratio constantly declined between F0 and F40 in the “Fig”, “Mirabel” and “Banana” groups but remained stable in the “Peach” group (Supplementary Figure S3D).

### 3.3. Females of Individual Lines

**Figure 4:**
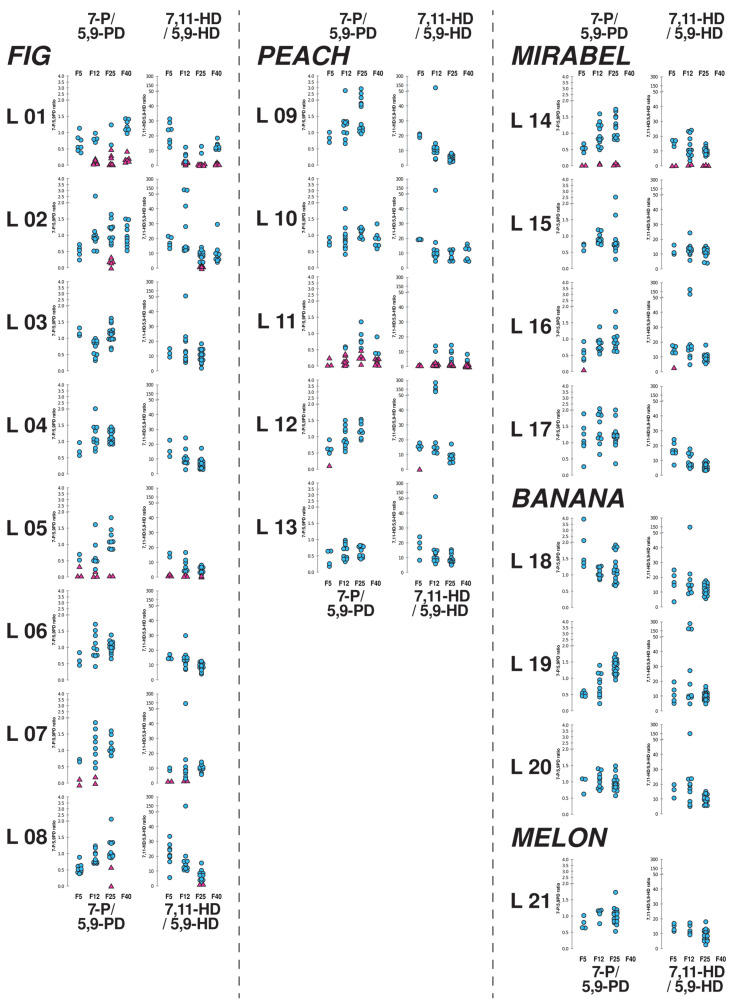
Distribution of two CHC ratios in females of all lines across generations. For each line, and at each generation (F5, F12, F25, F40; indicated above and below the global data set), we analysed two CHC ratios in individual 4-day-old females: 7-pentacosene/5,9-pentacosadiene (7-P/5,9-PD on the left), and 7,11-heptacosadiene/5,9-heptacosadiene (7,11-HD/5,9-HD, on the right). Individual females with temperate-like CHC profiles are represented as cyan circles; females with tropical-like CHC profiles are shown as magenta triangles. For each ratio, we used a different scale with a semi-logarithmic scale for the extreme higher values. These distributions correspond to the data shown in Table 2. For a more complete CHC data set, see Supplementary Figure S4.

We focused our analysis on four CHCs: 5,9-PD, 7-P, 7,11-HD and 5,9-HD, and calculated the 7-P/5,9-PD ratio and 7,11-HD/5,9-HD ratio (Figure 3 and Figure 4). For the sake of clarity, we only provide the mean of absolute CHC amounts or of the CHC ratio (individual CHC amounts are shown in the Supplementary Figure S4).

At F5, fifteen lines showed only the standard “temperate-like” female CHC pattern. These flies produced much more 7,11-HD than 5,9-HD (808 ng and 53 ng, respectively). On average, they also produced 116 ng 5,9-PD and 91 ng 7-P (Table 2; lines #1–4, 6, 8–10, 13, 15, 17–21). Their mean 7-P/5,9-PD ratio and 7,11-HD/5,9-HD ratio were 0.9 and 17.5, respectively.

The other six lines produced at least one female with a tropical-like CHC profile (lines #5, 7, 11, 12, 14, 16). Line #11 (“Peach 3”) produced 3/3 variant females. In total, 12 females (out of 29 females studied) showed a tropical-like CHC profile. The mean profile of these tropical-like CHC females consisted of 54 ng 7-P, 451 ng 5,9-PD, 546 ng 7,11-HD and 380 ng 5,9-HD. Their mean ratios of 7-P/5,9-PD and 7,11-HD/5,9-HD were 0.2 and 3.7, respectively.

At F12, five lines had tropical-like CHC females: this included lines #5, 7, 11, 14, which produced variant flies at F5, and the line #1, where 6/10 tropical-like CHC females appeared at F12. Line #11 (“Peach 3”) had 8/10 tropical-like CHC females at F12.

At F25, females with a tropical-like CHC profile were detected in six lines (#1, 2, 5, 8, 11, 14). Their frequency was very high in lines #1 (7/9) and #11 (5/10). Tropical-like CHC females also appeared in F25 at a high frequency in line #2 (5/16), and at lower frequency in line # 8 (2/12). In summary, tropical-like females were observed in lines #5, 11 and 14 at F5, F12 and F25.

Our F40 analysis focused on lines #1, 2, 10, and 11. The line #1 (“Fig 1”) and the line #11 (“Peach 3”) produced tropical-like CHC females with a high frequency (Table 2; 6/13 and 7/10, respectively) while lines #2 and 10 showed none. The mean 7-P/5,9-PD and 7,11-HD/5,9-HD ratios in tropical-like CHC females were 0.19 and 0.81 in the “Fig 1” line and 0.13 and 0.38 in the “Peach 3” line, respectively.

We analysed both the C25 ratio (7-P/5,9-PD) and C27 ratio (7,11-HD/5,9-HD) across generations (Figure 4). From F5 to F25 (or F40), the C25 ratio often showed a wide variability with stable or increasing mean values, whereas the C27 ratio often showed decreased mean values and a narrower variability. A significant regression across generations for the C25 ratio was only found in the Fig “fruit lines” with no tropical-like CHC females (Table 3). A significant change to a lower C27 ratio across generations was found in three of the four “fruit lines” that had no tropical-like profiles. The only exception was in the banana lines, which was also the only group without tropical-like CHC profiles. No effect was found in the lines with tropical-like CHC females.

**Figure 5:**
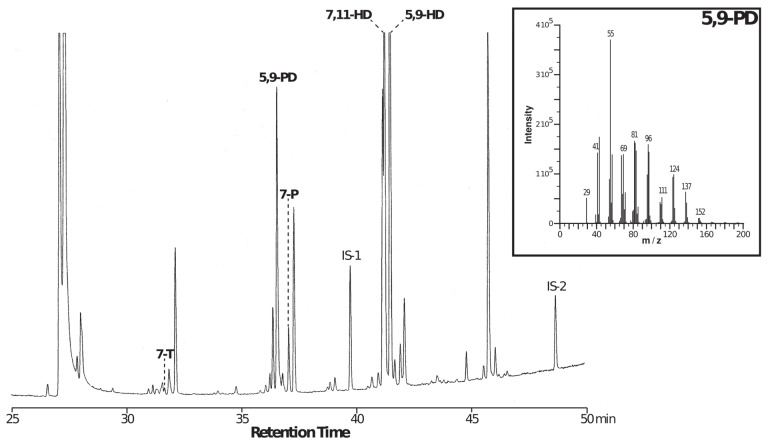
Typical GC-MS characterization of a single female. A single “Peach 3” female (at F40) was analysed using mass spectrometry coupled with gas chromatography. The whole GC profile is shown, including the five CHCs measured in this study, with increasing retention time from left to right: 7-tricosene (7-T), 5,9-pentacosadiene (5,9-PD), 7-pentacosene (7-P), 7,11-heptacosadiene (7,11-HD), 5,9-heptacosadiene (5,9-HD). The inset on the top right shows the ionic fragmentation spectra corresponding to 5,9-PD (see Section 2).

We also performed a GC-MS analysis to ascertain compound identity in tropical-like females. The ion fragmentation profile indicated that one of the most abundant compounds identified in tropical-like females was 5,9-PD (Figure 5), although 5,9-HD was also abundant. Out of a total of five females tested by GC-MS for each line, three “Fig 1” and five “Peach 3” females showed a tropical-like CHC profile, while the two other lines showed none. Therefore, with GC and GC-MS data pooled, “Fig 1” and “Peach 3” lines, respectively, produced a total of 9/18 (50%) and 12/15 (80%) tropical-like CHC females at F40.

### 3.4. Analysis of other CHCs in Males

Given the high levels of 5,9-PD and 5,9-HD in “Fig 1” and “Peach 3” females—both these compounds are normally observed in female flies from tropical regions—we checked the amounts of additional CHCs in “Fig 1” and “Peach 3” males at F25 and F40. In particular, we measured 7-P (high in West African males [41]) and 5-tricosene (5-T; high in Zimbabwe males [40]). At F25, the amounts of 5-T and 7-T were 1166 and 85 ng, respectively, in “Fig 1” males, and 1419 and 123 ng in “Peach 3” males. At F40, the 7-T and 7-P amounts were 1586 and 380 ng, respectively, in “Fig 1” males, and 1808 and 175 ng in “Peach 3” males. These levels were similar to those found in males from temperate regions showing standard CHC profiles.

## 4. Discussion

The principal aims of the present study were to (1) determine natural CHC variability in *Drosophila melanogaster* flies collected in a temperate region subjected to global warming (https://www.infoclimat.fr/climatologie/globale/dijon-longvic/07280.html, accessed on 28 March 2024) and (2) measure the effect of laboratory acclimation on their CHCs. We also investigated if there was a relationship between these two effects and the fruit preference of each line, as measured by its entry into the fruit-baited trap in the wild. Our *Drosophila* CHC sampling can be seen as the latest step in a series carried out on larger scales, beginning nearly three decades ago [36,41,42]. We obtained an “instant” picture of CHC diversity in space and time by collecting flies in a very limited area (400 sq. meters) and during a brief time window (3 h on 2 successive days). We evaluated the impact of global warming on CHCs by comparing our results with CHC profiles obtained in previous studies in the same area [42]. This study was prompted by reports of rapid adaptation of CHCs to changing environmental conditions [3,48,49]. In this sense, CHC variation can be considered, in part, to reflect insect adaptation to changing climatic conditions and diet [2,29,50]. Our approach was different to that involving the comparison of lines from a single parental laboratory strain raised on different food sources over many generations [51,52].

In our initial screening of F0 males, *c*Va did not vary between lines, unlike 7-T and 7-P. However, after one generation in the laboratory, all three pheromones varied between lines. In particular, all F1 “Fig lines” showed a lower 7-T/7-P ratio compared to the other lines (except line #9; Supplementary Figure S1D; Figure 3D). F12 males showed a general quantitative decrease which is difficult to interpret. At this generation, only the 7-T/7-P ratio—not affected by the overall decrease—revealed substantial inter-line differences. At F25 and F40, a marked variation was observed between most lines, for all parameters. The four “Fruit lines” groups we studied also showed differences: the 7-T/7-P ratio decreased faster across generations in “Banana lines” compared to “Peach lines”. Moreover, no variant CHC female was detected in “Banana lines”, unlike the other three “Fruit lines” groups (Table 2).

The high levels of *c*Va, 7-T and 7-P found in F0 and F1 males drastically decreased in F12 males (Supplementary Figures S2 and S3), for unknown reasons. Compared to F12, most F25 males showed stable or moderately increasing *c*Va and 7-T levels, while 7-P increased more, sometimes reaching a level close to that of F0/F1 males. When compared to F0/F1 males, most F40 lines showed either similar (*c*Va and 7-T) or higher levels (7-P) of the three pheromones. The greater increase in 7-P compared to 7-T probably explains the decreased 7-T/7-P ratio in most F40 males, if compared to earlier generations. Across generations, very similar patterns of variation were found for each parameter in the four “Fruit lines” groups except in the “Fig” group, which showed no 7-T/7-P ratio variation. The increase in 7-P levels that was frequently observed (leading to a decreased 7-T/7-P ratio) may reflect the rapid adaptation of male CHCs to constant warmer laboratory temperatures (25°) compared to outdoor temperatures (which varied between 13° and 22° [night/day], in Dijon at the beginning of September 2020). This hypothesis is supported by an experimental temperature shift (18°/25°) in early adult life which induced a lower 7-T/7-P ratio in control *D. melanogaster* males raised at 25° compared to males raised at 18° [5]. A global survey of these two compounds showed a relationship between the variation of the 7-T/7-P ratio and climatic variables [49].

Our field sampling revealed the presence of females with an unexpected CHC profile, mostly characterized by high levels of 5,9-pentacosadiene (25C; 5,9-PD) and 5,9-heptacosadiene (27C; 5,9-HD). 5,9-PD is a major pheromone in *D. serrata* flies, a species endemic to Australia (*D. montium* subgroup [53]). Previous studies of 7,11-HD-rich *D. melanogaster* females have not detected 5,9-PD [5,34,54] (Figure 1B) or did not identify the pentacosadiene isomer, which was generally very low [30,35,55]. In the only report identifying 5,9-PD in 7,11-HD-rich females, the level was very low ([45]; compound #33 in Figure 1). Studies of 5,9-HD-rich females (West African and/or Caribbean strains) have not detected 5,9-PD [30,55] or did not measure its level [37]. It has only been observed at a substantial level in Zimbabwe females ([40]; compound H in Figure 4). The CHC profiles of variant females reported in these studies persisted for years or even decades in constant laboratory conditions, indicating a clear genetic basis. For example, the stability of the 7,11-HD/5,9-HD ratio in the laboratory is linked to the expression of the *desat1* and *desat2* genes [33,34,39,56,57,58] (Figure 1A).

The presence of this novel “tropical-like” CHC variant profile might represent a rapid adaptation to the increased temperature that has occurred in temperate areas over the last few decades, or this profile might have come from flies brought by trade from tropical areas, or even following escape from a laboratory such as our own. A rapid adaptation to climate change has been observed for chromosome inversion polymorphisms in several Drosophila generalist species [59] and may coincide with the loss of genetic diversity [15,60]. Inadvertent variant fly importation is also possible; it is thought that ancestral afrotropical forms spread to the Americas through the slave trade [61,62], thus explaining the presence of 5,9-HD rich females in the Caribbean [36,38,55]. It seems unlikely that the tropical-like CHC females we observed originated from Zimbabwe populations, given the strong disadvantage experienced by Zimbabwe females in mixed cultures with 7,11-HD-rich females [39]. Furthermore, neither “Fig 1” or “Peach 3” males (lines with tropical-like CHC females) produced 5-T levels as high as those in Zimbabwe males [40,63] (Figure 1B). It is possible, however, that the tropical-like CHC flies originated from West Africa, where females show low 5,9-PD and high 5,9-HD levels. Over the generations, 5,9-PD levels could have progressively increased in surviving flies while 5,9-HD levels decreased, as an adaptation to a “warm temperate” climate. Male adaptation to warm temperate areas could have also led to a decrease in 7-P (compared to the high 7-P level found in tropical males). This is the case of “Peach 3” males (#11 line) which showed the lowest 7-P (and highest 7-T/7-P; Supplementary Figure S1C,D). It seems probable that such variants are due to commercial importation, but we cannot exclude either an escape from a laboratory or, less likely given the time scales, a spontaneous mutation either in a structural or a regulatory gene.

Carbon chain length variation in some CHC species represents an adaptation to changed temperatures [5,17,64]. This is produced by the altered activity of elongase enzymes involved in the addition of two carbons on even-numbered carbon chains before the final decarboxylation step that produces odd-numbered carbon chains [25,29,30,65,66] (Figure 1A). This role of an elongase has been described in the biosynthesis of the major CHC pheromone in the domestic fly *Musca domestica* [67]. We propose that the variant CHC females we report here originated from tropical females with low 5,9-PD and high 5,9-HD profiles, inadvertently imported through commercial routes. These particular genes have spread in the local populations, enabling them to progressively adapt to the effects of global warming.

We could not evaluate the field frequency of this variant CHC phenotype because F0 females were not tested, as this would have killed them before they could lay sufficient eggs to establish the lines. However, its occurrence in 9/21 strains (with all generations pooled) indicates that it was initially present at a low but not rare frequency. Over the generations spent in the laboratory, lines with variant CHC females evolved in different ways. The variant CHC profile persisted (lines #1, 5, 11, 14; Table 2), sometimes at high frequencies, while sampling variation led to its “appearance” in two lines (#2, 8) and “disappearance” from three lines (#7, 12, 16). Overall, our acclimation data suggest that variant CHC flies had no fitness disadvantage in mixed culture with standard native flies (such as the “Peach 3” line). Furthermore, the high frequency at F25 of variant flies in line #2 suggests that genes underlying this profile—maybe in interaction with the genetic background of this line—provided a fitness advantage. Further sampling in the same area would allow us to determine whether the frequency of 5,9-PD-rich flies has increased relative to native flies, perhaps leading to an “invasive” *D. melanogaster* hydrocarbon phenotype.

The C25 ratio (7-P/5,9-PD) and C27 ratio (7,11-HD/5,9-HD) have differently evolved during laboratory acclimation according to the presence, or not, of tropical-like CHC females in the “Fruit lines” (Figure 4). These data indicate that 5,9-HD increased at the expense of 7,11-HD. This also suggests that genes other than *desat2* are involved in the C27 ratio and increased their frequency during acclimation [39]. Across generations, the C25 ratio often maintained high variability and showed higher median values. This indicates that the production of 7-P and of 5,9-PD are partly dissociated: in the laboratory, 7-P increased faster than 5,9-PD. In constant warmer laboratory conditions, a decline in 5,9-PD levels could be beneficial to 5,9-HD, thus explaining the reciprocal variation of the two ratios (C25 increased while C27 decreased) shown by many lines across generations. An apparent bimodal distribution (or at least a clear-cut separation) between standard and variant ratio values was observed in lines #1, 5, 14 for the C25 ratio and only in #1 line for the C27 ratio. Taken together, these data indicate that under laboratory conditions, female production of 7-P generally increased, as in males. This was probably due to the increased activity of an elongase. A similar enzymatic process can also be presumed to lie behind the increase in 5,9-HD at the expense of 5,9-PD.

In conclusion, in a field survey we found *D. melanogaster* flies with novel CHC female profiles which subsequently evolved as the strains acclimated to laboratory conditions. The survival of flies with such CHC profiles in temperate regions may be due to climate change. More generally, sampling insect CHCs in a limited geographic area can provide sensitive markers of climate change, on condition that the insects are studied before they acclimate to laboratory conditions.

## Figures and Tables

**Table 1:** General characteristics of 21 freshly caught lines. A variable number of F0 female and male flies was caught in traps baited with different types of fruit (fig/peach/mirabel/banana/melon). Each line was given a number (#). In some traps, we caught and discarded non-*D. melanogaster* flies: line #15: one *D. virilis* female; line #16: one *D. suzukii* male; line #21: one *D. suzukii* female; line #22 “Melon2”: one *D. virilis* female and one *D. virilis* male and *D. busckii* flies. In each line, F2 males were crossed to control Cs females to ascertain species identity based on the presence of F3 female and male progeny. The proportion of F3 progeny was measured and the number of F3 males counted. Intra-line lethality was measured in 4-day-old females and males kept in small groups of 5 flies (at F12 and F25 generations pooled). For each sex, the numbers shown on the left and right sides represent the dead and live flies, respectively.

	Line	Number of F0 Flies		F3 Progeny	F3 Male	Adult Lethality (F12 & F25)			
“Fruit Lines”	#	Female	Male	Females/Males	Progeny	Female		Male	
Fig 1	#1	10	8	60/40	102	0	29	0	50
Fig 2	#2	9	4	60/40	107	7	40	5	80
Fig 3	#3	7	6	50/50	97	13	53	0	60
Fig 4	#4	12	8	50/50	101	1	42	1	45
Fig 5	#5	10	10	50/50	40	5	34	0	61
Fig 6	#6	10	6	50/50	73	2	38	2	75
Fig 7	#7	9	9	50/50	84	0	22	5	62
Fig 8	#8	7	11	50/50	79	6	40	4	68
Peach 1	#9	5	10	50/50	32	7	39	1	70
Peach 2	#10	11	10	50/50	95	10	31	1	53
Peach 3	#11	6	11	55/45	89	4	34	0	85
Peach 4	#12	2	3	55/45	60	5	27	3	60
Peach 5	#13	5	4	50/50	31	5	37	1	55
Mirabel 1	#14	5	6	50/50	72	1	40	3	70
Mirabel 2	#15	4	5	55/45	110	7	29	1	54
Mirabel 3	#16	5	1	60/40	83	5	30	5	60
Mirabel 4	#17	4	6	45/55	108	7	31	3	69
Banana 1	#18	5	7	50/50	105	1	42	1	65
Banana 2	#19	1	1	45/55	80	4	36	0	65
Banana 3	#20	2	5	45/55	51	5	34	3	64
Melon 1	#21	3	3	55/45	46	3	34	2	60
Melon 2	#22	3	2						

**Table 2:** Distribution of female CHC profiles across generations. In each line and generation, we counted the number of female flies showing a standard CHC profile (high 7,11-HD/low 5,9-HD) or a variant CHC profile (high 5,9-PD and 5,9-HD/low 7,11-HD). CHC profiles were analysed using GC. At F40, we analysed varying numbers of flies in 4 lines using GC and 5 flies with GC-MS (data shown in brackets). Some flies showing a relatively high level for only one 5,9-diene but not for the other were not considered as variant CHC flies (see Figure 3 and Figure S3).

	F5		F12		F25		F40	
	Phenotypes		Phenotypes		Phenotypes		Phenotypes	
Line #	Temperate-like	Tropical- like	Temperate- like	Tropical- like	Temperate- like	Tropical- like	Temperate- like	Tropical- like
#1	7		4	6	2	7	7 (+2)	6 (+3)
#2	5		10		11	5	10 (+5)	
#3	3		10		15			
#4	3		10		15			
#5	2	3	7	3	9	2		
#6	3		10		13			
#7	2	2	8	2	8			
#8	9		10		10	2		
#9	3		10		12			
#10	3		10		8		7 (+5)	
#11		3	2	8	5	5	3	7 (+5)
#12	4	1	10		8			
#13	4		10		12			
#14	4	2	13	2	12	3		
#15	3		10		11			
#16	5	1	10		11			
#17	7		10		13			
#18	5		9		15			
#19	5		10		17			
#20	3		10		15			
#21	4		5		14			

**Table 3:** Regression analysis for two ratios in females of the “Fruit lines” groups according to the generation. Fruit lines, grouped according to the fruit bait they were caught in, were separated into two categories: Lines producing at least one female with temperate-like CHC profile (on the left panel) and lines with no temperate-like CHC females (at any generation; on the right panel). For each group, we show the regression slope for the 7-P/5,9-PD and 7,11-HD/5,9-HD ratios (dependent variables) versus generations (as explanatory variable), and the corresponding *p*-values. Significant values were only found for “Fruit lines” with temperate-like CHC females.

	Temperate-like				Tropical-like			
	7-P/5,9PD		7,11-HD/5,9-HD		7-P/5,9PD		7,11-HD/5,9-HD	
	Slope	*p*	Slope	*p*	Slope	*p*	Slope	*p*
Fig	0.014	0.0001	−0.540	0.015	0.003	0.128	−0.017	0.066
Peach	0.003	0.469	−1.006	0.006	−0.001	0.783	−0.109	0.069
Mirabel	−0.007	0.346	−0.153	0.298	0.011	0.033	−0.484	0.051
Banana	0.007	0.252	−1.138	0.048				
Melon	0.007	0.273	−0.219	0.009				

## Data Availability

An xlsx file containing all raw data will be available as Supplementary Materials.

## Supplementary Materials

The following supporting information can be downloaded at: https://www.mdpi.com/article/10.3390/insects15040273/s1, Figure S1: Pheromone production in males of all lines over five generations; Figure S2: Male pheromonal production in each line across generations; Figure S3: Male pheromonal production in each group of “Fruit lines” across generations; Figure S4: Distribution of CHCs in females of all lines across generations.
