# Supplementary material for: Natural Diversity of Cuticular Pheromones in a Local Population of Drosophila after Laboratory Acclimation
Source: Insects. 2024 Apr 15;15(4):273. doi: 10.3390/insects15040273 (PMC11050499; doi:10.3390/insects15040273)
Supplement: Supplementary file 1 [file insects-15-00273-s001.zip › insects-2924678-supplementary.pdf]

SUPPLEMENTAL FIGURES

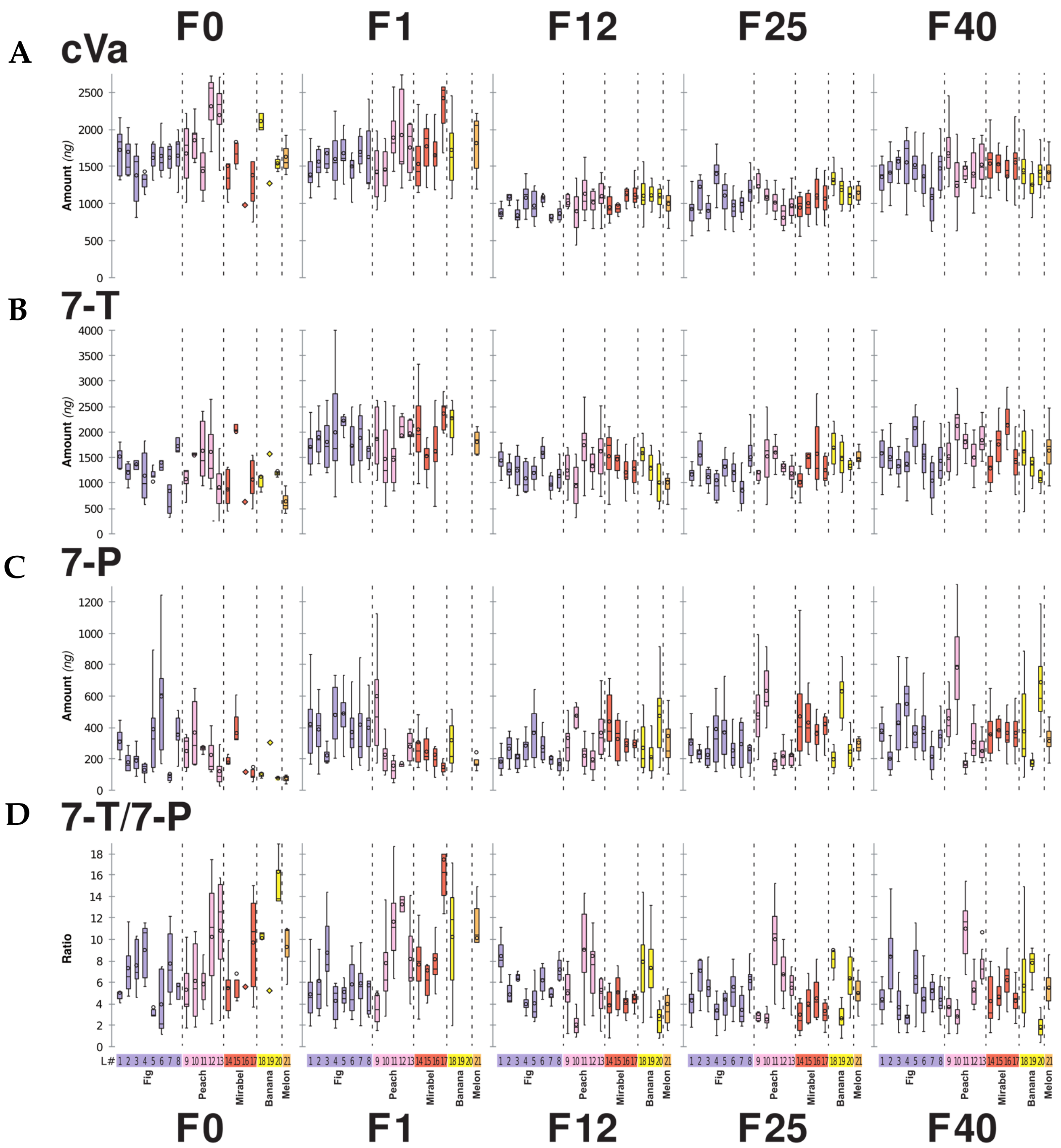

Supplemental Figure S1. Pheromone production in males of all lines over five generations.

We analysed the amounts of (**A**) *c*Va, (**B**) 7-T, (**C**) 7-P (all indicated in ng) and (**D**) the 7-T/7-P ratio in individual 4-day old male flies either caught in nature (F0) or after 1, 12, 25 and 40 generations in the laboratory (F1, F12, F25, F40, respectively; from left to right). Lines are shown with respect to their initial fruit preference, as measured by the trap they were found in (indicated below the global data set and separated by dotted vertical lines), and according to their line # (shown below). For F0: N=3-

6 (except lines #16 and 19: N=1); F1: N=9-15 (except lines #3, 8, 12, 13, 17, 21: N=5; no data for lines #19 and 20); F12: N=10; F25: N=9-13; F40: N=15-20.

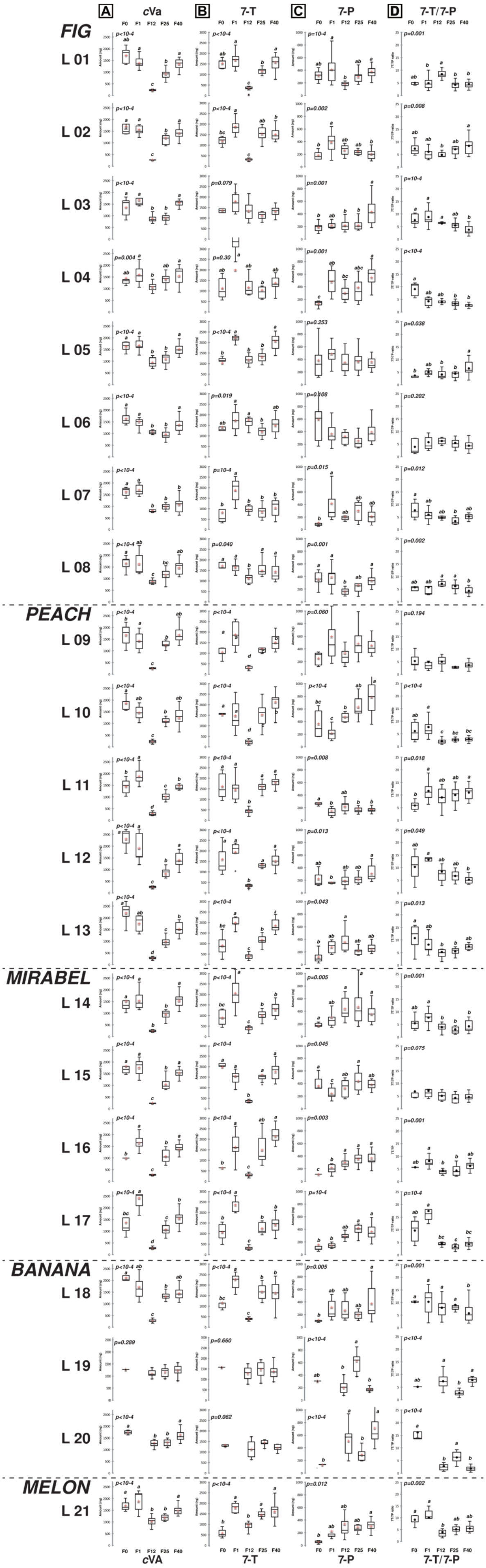
A cVa
B 7-T
C 7-P
D 7-T/7-P
F0 F1 F12 F25 F40
FIG
L 01
L 02
L 03
L 04
L 05
L 06
L 07
L 08
PEACH
L 09
L 10
L 11
L 12
L 13
MIRABEL
L 14
L 15
L 16
L 17
BANANA
L 18
L 19
L 20
MELON
L 21
cVA
7-T
7-P
7-T/7-P

Supplemental Figure S2. Male pheromonal production in each line across generations.

For each line, we analysed the level of (**A**) cVa, (**B**) 7-T, (**C**) 7-P (all indicated in ng) and (**D**) the 7-T/7-P ratio in individual 4-day old male flies of each line (# shown on the left) across F0, F1, F12, F25, F40 (indicated above bars). These data correspond to reorganized data shown on Supplemental Figure 1. Different letters indicate significant differences between different generations. For more details on parameters and statistics, see Figures 1 & 3 legends.

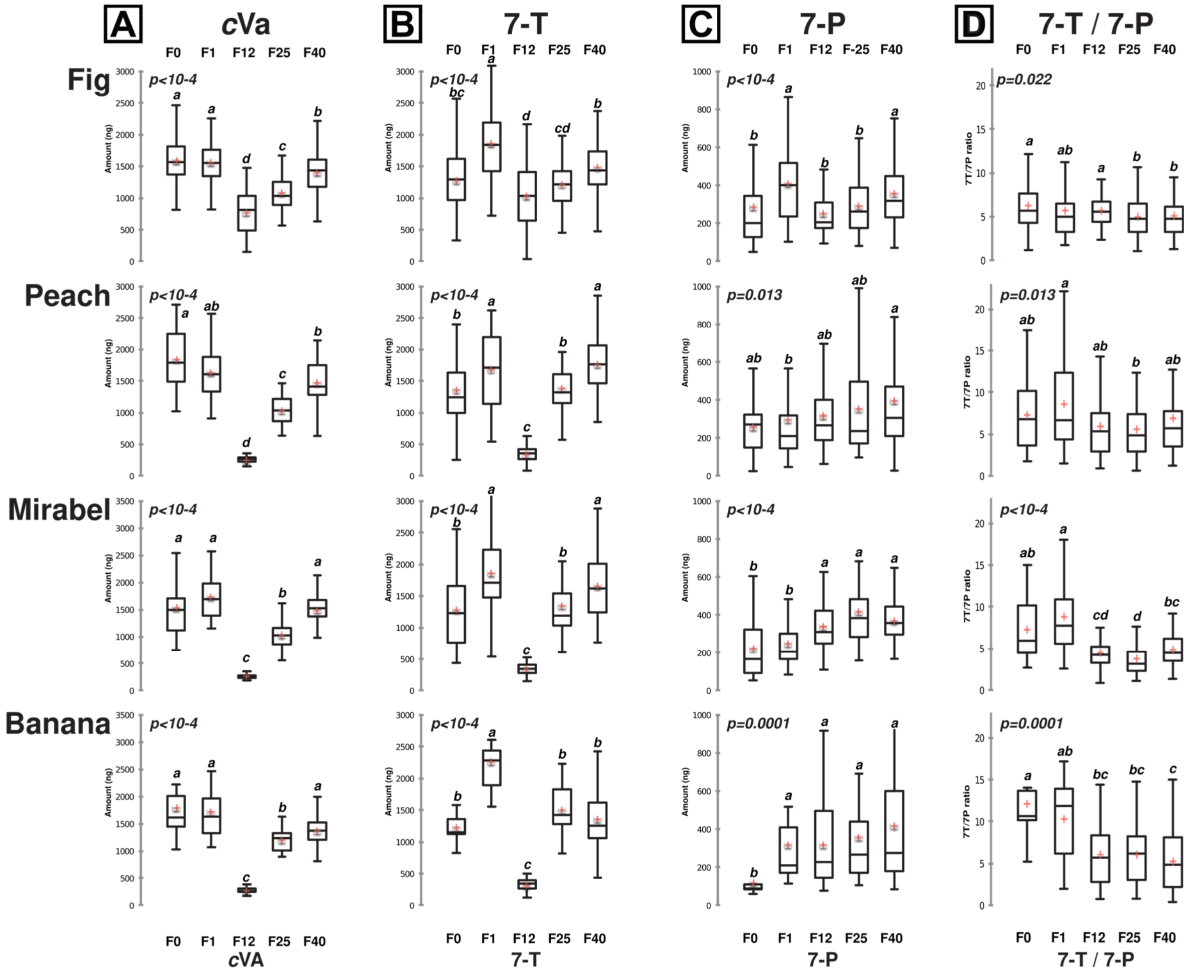

Supplemental Figure S3. Male pheromonal production in each group of "Fruit lines" across generations. For each pheromonal parameter: (**A**) cVa, (**B**) 7-T, (**C**) 7-P and (**D**) the 7-T/7-P ratio, lines were grouped into "Fruit lines" according to their initial fruit preference. Measures were taken at F0, F1, F12, F25 and F40 (shown from left to right). These data correspond to differently organized data shown on Figure 3. For more details on parameters and statistics, see legends of Figures 1 & 3 and of Supplemental Figure 1.

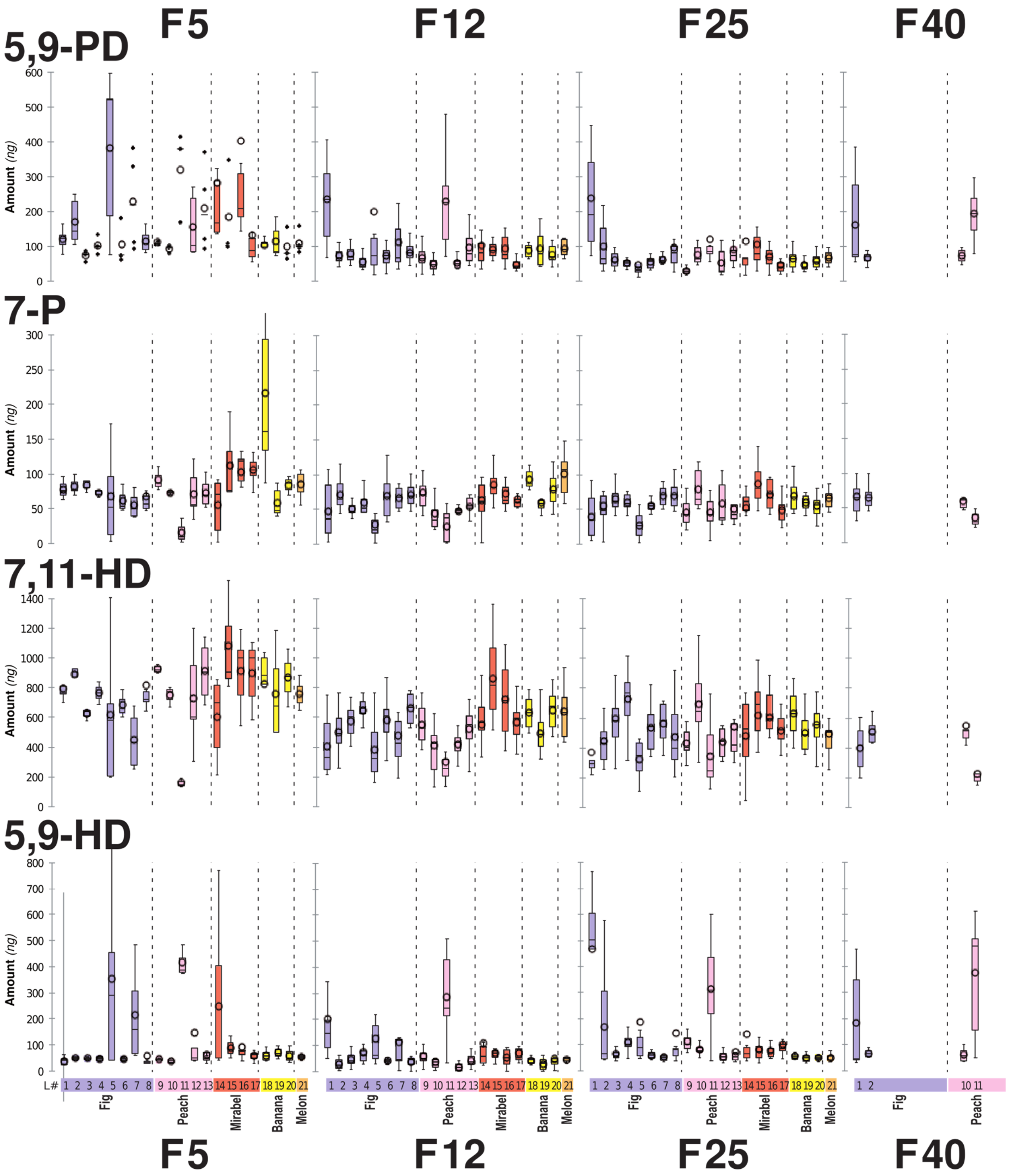

Supplemental Figure S4. Distribution of CHCs in females of all lines across generations.

For each line, and at each generation (F5, F12, F25, F40), we measured the level of four CHCs in individual 4-day old females. CHCs analysed were, from top to bottom: 5,9-pentacosadiene (5,9-PD), 7-pentacosene (7-P), 7,11-heptacosadiene (7,11-HD) and 5,9-heptacosadiene (5,9-HD). Data are shown in a composite manner: with box plots when N ≥5 and with dots for individual values. Bars and dots are filled in cyan and magenta corresponding to temperate-like or tropical-like CHC profiles, respectively. These data were used to calculate the two ratio (shown on Figure 4). For more details on parameters and statistics, see Figures 3 & 4 legends.
